# Fatal Outcome of Suicidal Multi-Substance Ingestion Involving Sodium Nitrate and Nitrite Toxicity: A Case Report and Literature Review

**DOI:** 10.15388/Amed.2025.32.1.11

**Published:** 2025-02-18

**Authors:** Sofija Saulė Kaubrytė, Sigitas Chmieliauskas, Giedrė Salyklytė, Sigitas Laima, Diana Vasiljevaitė, Jurgita Stasiūnienė, Paulius Petreikis, Robertas Badaras

**Affiliations:** 1 Faculty of Medicine, Vilnius University, Vilnius, Lithuania; 2 Department of Pathology, Forensic Medicine, Institute of Biomedical Sciences, Faculty of Medicine, Vilnius University, Vilnius, Lithuania; 3 Department of Anatomy, Histology and Anthropology, Institute of Biomedical Sciences, Faculty of Medicine, Vilnius University, Vilnius, Lithuania E-mail:; 4 Centre for Toxicology, Clinic of Anaesthesiology, Reanimatology and Critical Care Medicine, Institute of Clinical Medicine, Faculty of Medicine, Vilnius University, Vilnius, Lithuania E-mail: ORCID ID

**Keywords:** nitrate, nitrite, poisoning, suicide, nitratai, nitritai, apsinuodijimas, savižudybė

## Abstract

**Background:**

Nitrate and nitrite toxicity, particularly from sodium nitrite and sodium nitrate ingestion, has become a critical public health issue due to its role in accidental poisoning, foodborne exposure, and intentional self-harm. Sodium nitrite, commonly used in food preservation, is increasingly linked to suicide, with online resources providing accessible information on lethal dosages. This trend underscores an urgent need for regulatory action and preventive strategies. This report details a fatal case of nitrate and nitrite toxicity in a 19-year-old female, presenting a complex toxicological profile involving ethyl alcohol, amphetamines, and additional pharmaceuticals.

**Materials and methods:**

A systematic literature search was conducted across *PubMed* and *Google Scholar* databases, spanning articles published over a period of the last 10 years, utilizing keywords relevant to the topic under consideration and their combinations. 58 pertinent articles were selected, supplemented by data from the *Lithuanian State Forensic Medicine Service*, involving a clinical case. Autopsy findings, toxicological analyses, and contextual details were meticulously examined to elucidate the mechanism and circumstances of death.

**Case presentation:**

A 19-year-old female was found deceased in her home, alongside a suicide note indicating intent to self-harm through ingestion of sodium nitrate, sodium nitrite, and multiple medications. The autopsy findings included cherry-brown lividity, dark, non-coagulated blood within the heart chambers, and significant multi-organ congestion, consistent with methemoglobinemia and systemic hypoxia. Toxicology results confirmed a blood alcohol concentration of 1.22‰, with trace levels of amphetamine, atropine, and quetiapine, while nitrites detected in the gastric contents confirmed sodium nitrite as a primary toxic agent. The toxic synergy of these substances ultimately led to fatal multi-organ compromise.

**Conclusions:**

This case emphasizes the serious risks associated with nitrate and nitrite ingestion, particularly in instances of intentional overdose facilitated by readily accessible online information on lethal dosages. The autopsy findings reveal characteristic signs of methemoglobinemia and systemic hypoxia; however, a thorough forensic assessment must also consider additional factors, including the presence of a suicide note, the scene context, and any substances collected at the location.

**Lessons:**

Due to the inherent instability of nitrite in blood, its post-mortem detection is often challenging, thereby complicating toxicological confirmation. The increasing accessibility and misuse of sodium nitrite underscore an urgent need for regulatory oversight, public awareness, and enhanced preventive measures to address the rising incidence of intentional sodium nitrite toxicity, particularly within at-risk populations.

## Abstract (it)

**Įvadas:**

Nitratų ir nitritų toksiškumas siejamas su žmogaus sveikatai žalingu poveikiu, pastebimu atsitiktinių apsinuodijimų atvejais, vartojant nitratais užterštą geriamąjį vandenį ar netinkamai panaudojus natrio nitrito druską maistui ruošti. Pastaruoju metu visuomenėje pastebima tyčinių apsinuodijimų nitratais ir nitritais dažnėjimo tendencija. Interneto šaltiniuose pateikiama aprašymų apie galimą natrio nitrito, maisto pramonėje naudojamo konservanto, pasitelkimą suicidiniais tikslais. Susirūpinimą kelia virtualiose paieškos platformose lengvai prieinama informacija apie savižudybės metodų instrukcijas, taikant mirtinas nitratų ir nitritų dozes, todėl, siekiant sumažinti pavojingus visuomenės sveikatai padarinius, pabrėžtinas reguliacinių priemonių ir prevencinių strategijų poreikis. Straipsnyje pateikiamas atvejo aprašymas, iliustruojantis mirtiną apsinuodijimą, susisijusį su nitratų ir nitritų toksiškumu bei pasireiškusį sudėtingo toksikologinio profilio fone, mirusiosios kraujyje taip pat aptikta etilo alkoholio, amfetamino ir kitų vaistų pėdsakų.

**Medžiaga ir metodai:**

Pasitelkus temą atitinkančius raktinius žodžius ir jų derinius atlikta mokslinės literatūros paieška *PubMed* ir *Google Scholar* duomenų bazėse. Atitinkamai išanalizuoti 58 viso teksto straipsniai ir santraukos, publikuoti anglų kalba per pastaruosius 10 metų. Remiantis Lietuvos valstybinės teismo medicinos tarnybos duomenimis, aprašytas apsinuodijimą nitratais iliustruojantis klinikinis atvejis. Mirties priežasčiai ir aplinkybėms išsiaiškinti išsamiai nagrinėtos autopsijos išvados, analizuoti toksikologinių tyrimų rezultatai bei įvertinti papildomi su atvejo kontekstu susiję įrodymai.

**Rezultatai:**

19 metų merginos lavonas buvo rastas kambaryje, šalia aptiktas atsisveikinimo raštelis, taip pat išsiaiškinta apie galimą natrio nitrito ir natrio nitrato pavartojimą prieš mirtį, todėl, atsižvelgus į surinktus įrodymus, įtarta savižudybė. Autopsijos metu nugariniuose kūno paviršiuose buvo matomos išplitusios, rudai vyšninės, ryškaus intensyvumo lavondėmės, aptikta tamsaus, rudai vyšninės spalvos kraujo širdies ertmėse, nustatyta veninė vidaus organų pilnakraujystė, galvos smegenų ir plaučių pabrinkimas. Atlikus toksikologinį tyrimą, lavono kraujyje buvo nustatyta 1,22 ‰ etilo alkoholio koncentracija, taip pat rasta 0,022 mg/l amfetamino, 0,010 mg/l atropino ir 0,024 mg/l kvetiapino. Lavono skrandyje aptikta nitritų. Nustatyta mirties priežastis – ūminis suminis nitratų, narkotinių bei kitų stipriai veikiančių medžiagų toksinis poveikis.

**Išvados:**

Aprašytas atvejis atspindi nitratų ir nitritų toksiškumo problematiką, išryškėjančią tyčinių apsijuodijimų kontekste, kai suicidiniams motyvams įgyvendinti dažnai pasitelkiamos įvairios papildomos medžiagos ir vadovaujamasi instrukcijomis, paplitusiomis interneto šaltiniuose. Autopsijų radiniai intoksikacijų nitratais ir nitritais atvejais atspindi būdingus methemoglobinemijos ir sisteminės hipoksijos požymius, taip pat svarbios papildomos informacijos tiriant tokio pobūdžio apsinuodijimus suteikia laboratorinių tyrimų rezultatai ir mirties vietoje aptinkami įrodymai. Vis dėlto atliekant toksikologinį ištyrimą, kai įtariamas galimas apsinuodijimas nitratais ar nitritais, dažnai susiduriama su šių medžiagų nustatymo netikslumais, atsirandančiais dėl nitritų cheminio nestabilumo kraujo mėginiuose. Atsižvelgiant į nitratų ir nitritų toksiškumo keliamus iššūkius ir visuomenėje vis dažniau pasitaikančius su savižudybiniais motyvais susijusius apsinuodijimų atvejus, pabrėžtina sustiprintų prevencinių priemonių taikymo svarba, ypač nustatytose rizikos grupėse.

## Introduction

Nitrates, primarily sodium nitrate (NaNO_3_) and potassium nitrate (KNO_3_), are naturally occurring compounds found in soil and water, where they are absorbed by plants. These compounds accumulate in significant concentrations, particularly in vegetables such as spinach, lettuce, and beetroot, due to agricultural fertilization practices [1,2]. While nitrates are generally less toxic to humans, bacterial metabolism in the oral cavity and the gastrointestinal tract can convert a portion into nitrites (NO_2_^–^), which pose a higher toxicity risk and can lead to methemoglobinemia, especially in vulnerable populations such as infants [2,3,4].

Nitrites, such as sodium nitrite (NaNO_2_), are extensively used in food preservation to inhibit bacterial growth and maintain color stability [5,6]. However, sodium nitrite is also associated with significant toxicity, with both accidental and intentional ingestions reported to cause fatal methemoglobinemia, characterized by cyanosis and hypoxemia [7,8]. Emerging trends indicate a global increase in sodium nitrite-related suicides, with cases frequently linked to information shared on online forums. This troubling pattern has been observed in several countries, including the United States, Canada, Australia, Portugal, Slovakia, and France [9–18]. Younger individuals, particularly those with preexisting mental health issues, appear to be disproportionately affected, emphasizing the urgent need for enhanced preventive measures and regulatory oversight.

This report presents a fatal case of sodium nitrate and nitrite poisoning in a 19-year-old female investigated by the *Lithuanian State Forensic Medicine Service* in 2023. The case is characterized by the detection of nitrites in gastric contents and a clinical history indicating intentional ingestion of sodium nitrate, with suicide as the suspected intent. Additionally, toxicological analysis revealed the presence of amphetamine and a blood alcohol concentration of 1.22‰, contributing to a complex toxicological profile. Pathological findings included cherry-brown livor mortis, liquid chocolate-brown blood in the cardiac chambers, significant venous congestion of internal organs, and cerebral and pulmonary edema, consistent with fatal nitrite toxicity compounded by multi-substance ingestion.

## Methods

A comprehensive literature search was conducted using the *PubMed* database and the *Google Scholar* search engine to identify relevant studies on nitrate and nitrite toxicity. The keywords we used include ‘nitrate’, ‘nitrite’, ‘saltpeter’, ‘sodium nitrate’, ‘sodium nitrite’, ‘poisoning’, ‘intoxication’, and ‘suicide’. These terms were selected to capture a wide range of studies focusing on the toxicological, clinical, and forensic aspects of nitrate and nitrite exposure, particularly in cases of intentional self-harm. The search was limited to articles published in English within the last decade (2014–2024) to ensure the inclusion of recent and relevant data reflecting the contemporary trends in nitrite-related toxicity and its forensic implications. This timeframe was chosen to capture the emerging patterns, such as the increasing prevalence of sodium nitrite misuse in suicide cases, as documented in recent literature. A total of 58 pertinent articles were identified and included in the review. To enhance the understanding of global control measures, additional searches for official publications and regulatory documents were conducted to identify policies addressing sodium nitrite. For example, the European Commission’s Regulation (EU) 2023/2108, which reduces the maximum allowable nitrite levels in food products, was included to illustrate effective strategies for mitigating public health risks associated with sodium nitrite. Additionally, detailed information on the case was obtained from the Lithuanian State Forensic Medicine Service, including vital contextual details such as the incident location, the time of death, and the presumed cause of death. A complete autopsy was conducted to thoroughly investigate the circumstances of death. During the forensic dissection, blood and urine samples were systematically collected for toxicological analysis. Alcohol levels were determined by using the headspace gas chromatography technique, offering precise insights into the toxicological profile of the case. This method ensured reliable detection and quantification of ethanol and other substances, contributing to a detailed forensic analysis of the fatal outcome.

## Limitations

This case report highlights significant forensic and toxicological findings associated with sodium nitrite toxicity; however, the absence of direct measurement of methemoglobin (MetHb) levels represents a limitation. While findings such as cherry-brown discoloration, systemic hypoxia, and toxicological evidence strongly suggest methemoglobinemia, quantitative MetHb levels were not obtained during the investigation. MetHb concentration is critical for diagnosing and understanding the severity of nitrite-induced toxicity, as it provides direct evidence of the extent of oxygen transport impairment. The lack of this data limits the ability to quantitatively assess the severity of methemoglobinemia and its specific contribution to the fatal outcome. Future studies and forensic investigations would benefit from incorporating MetHb quantification to enhance the understanding of nitrite toxicity and improve the clinical and forensic interpretation of similar cases.

## Case presentation

The body of a 19-year-old female was discovered in her home, with no visible signs of external violence. A suicide note which was found at the scene indicated intentional self-harm. According to the ambulance report, the deceased had ingested sodium nitrate, sodium nitrite, and multiple medications before death and had experienced respiratory distress prior to succumbing. The mother confirmed her daughter’s intentional ingestion of sodium nitrate (possibly saltpeter), although the timing of ingestion was not determined.

External examination revealed a lean female of European descent, approximately 167 cm in height, with pronounced post-mortem lividity. The postmortem lividity (livor mortis), presenting as bright cherry-brown discoloration on the dorsal regions, suggested elevated methemoglobin levels consistent with nitrite exposure. It was fixed upon digital compression, indicating post-mortem stabilization. No external traumatic injuries were observed.

**Fig. 1:**
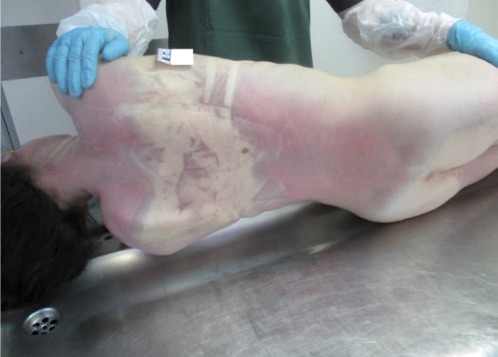
Fixed, cherry-brown livor mortis of bright intensity on the dorsal regions, remaining unchanged under digital compression [A photograph showing the dorsal regions of a deceased individual with distinct cherry-brown discoloration (livor mortis). The coloration is bright and remains unchanged when pressed with a finger, indicating fixed post-mortem lividity.]

**Fig. 2:**
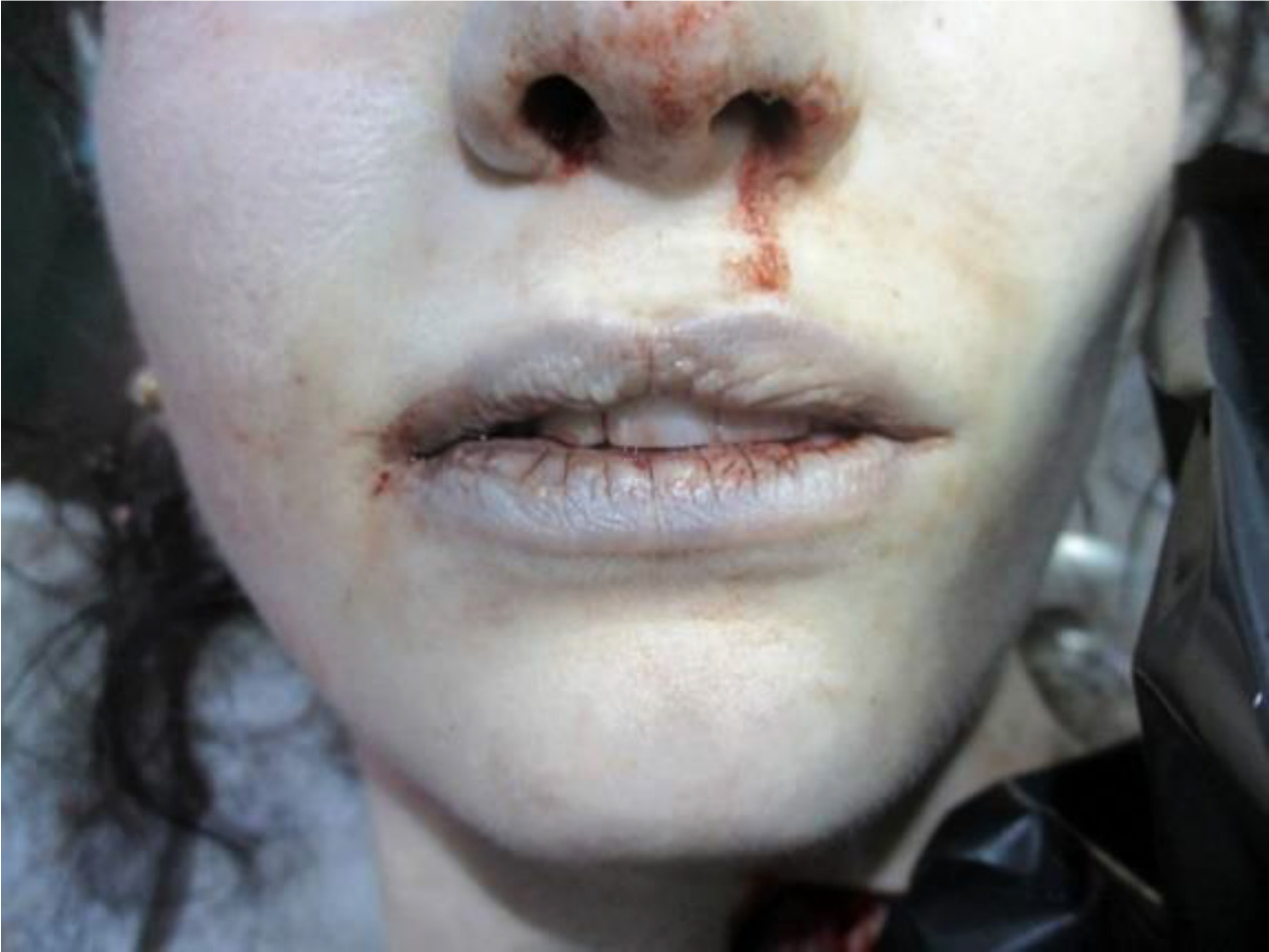
Cyanosis of the lips and skin pallor indicating tissue hypoxia, with blood staining around the nostrils suggestive of respiratory distress prior to death [A close-up photograph of a deceased person’s face, displaying bluish discoloration of the lips (cyanosis) and pale skin, indicative of oxygen deprivation. Blood is visible around the nostrils, suggesting respiratory distress before death.]

The autopsy findings revealed evidence of significant vascular congestion and hypoxia, consistent with sodium nitrite toxicity. The cardiac chambers contained liquid chocolate-brown blood, and the lungs exhibited marked vascular stasis, dark purple discoloration, and pulmonary oedema with fine foamy secretions. These findings are indicative of methemoglobinemia and systemic hypoxia. The brain showed substantial vascular congestion, flattened gyri, shallow sulci, and petechial haemorrhages, consistent with cerebral oedema. The stomach contained approximately 15 ml of a brown liquid, and nitrites were detected in the gastric contents, further corroborating sodium nitrite ingestion as a primary factor in the fatality.

During the forensic toxicology analysis, nitrites were detected in the stomach. The blood ethyl alcohol concentration was 1.22‰, indicating mild intoxication. Trace amounts of amphetamine (0.022 mg/L), atropine (0.010 mg/L), and quetiapine (0.024 mg/L) were also detected. No other toxic substances of high potency were identified in the toxicological analysis.

Collectively, these findings support the conclusion that the individual succumbed to the toxic effects of sodium nitrite ingestion, compounded by the synergistic impact of alcohol and other substances. The observed pathological and toxicological findings highlight the significant forensic implications of nitrite toxicity in intentional self-harm cases.

## Discussion

Nitrate and nitrite toxicity poses substantial health risks, particularly to vulnerable populations such as infants and adults exposed through drinking water or food [2–4]. Infants are especially susceptible to methemoglobinemia due to low cytochrome b reductase activity and oxidizable fetal hemoglobin, as demonstrated in cases linked to nitrate-rich dietary exposure during early childhood [4]. In adults, accidental ingestion of nitrites resulting from labeling errors or improper use has caused severe methemoglobinemia, emphasizing the need for stringent regulatory standards and accurate labeling. Incidents such as mislabeled sodium nitrite in food preparation and excessive use in homemade products underscore the dangers of unregulated nitrite handling and the importance of public safety measures [19–21]. Contamination of drinking water with nitrates exacerbates these risks and is associated with serious health outcomes, including infantile methemoglobinemia and congenital anomalies such as neural tube defects and oral clefts [1,3,22]. High nitrate levels in agricultural and industrial areas, such as southern Tehran and parts of the United States, frequently exceed safety standards, highlighting the urgent need for region-specific water quality regulations to protect public health [22–24].

Accidental exposure to nitrates and nitrites poses significant health risks due to their potential to induce methemoglobinemia, a condition characterized by cyanosis, hypoxia, and systemic effects. Diverse sources of exposure, such as traditional medicine preparations, mislabeled industrial chemicals, and nitrate-based supplements, highlight the need for improved safety practices and public education [25–27]. For instance, industrial accidents involving nitrite inhalation have resulted in fatal methemoglobinemia, underscoring the critical importance of workplace safety protocols [26]. These cases demonstrate the necessity of stringent regulatory standards so that to prevent accidental nitrate and nitrite exposure and improve public health outcomes. In addition to accidental exposure, the intentional misuse of alkyl nitrites, commonly referred to as poppers, presents significant health risks. Due to their oxidizing effects, these substances can lead to methemoglobinemia and, in rare cases, severe cardiac arrhythmias. Documented cases of acute toxicity include cyanosis, hypoxia, and seizures, which often require prompt intervention with methylene blue to mitigate the effects [28–32]. Public awareness campaigns targeting the recreational misuse of nitrites are crucial for reducing these risks and addressing the broader implications of nitrite toxicity.

Nitrite toxicity poses significant risks across various contexts, including accidental exposure, recreational misuse, and the alarming rise of sodium nitrite-related suicides. The increasing accessibility of sodium nitrite and its promotion through online platforms represent a significant and emerging public health concern. Recent epidemiological analyses reveal a notable global increase in sodium nitrite-related suicides, driven in part by the dissemination of detailed information about its use through online resources. For instance, data from the CDC’s National Vital Statistics System (2018 through July 2023) identified 768 suicides involving antidotes and chelating agents, including sodium nitrite, among 268,972 cases, representing less than 1% of all suicides but reflecting a rising trend linked to the compound’s increasing availability and online dissemination [9–11]. Online forums and e-commerce platforms facilitate the promotion of sodium nitrite for suicide, providing explicit guidance on its acquisition, dosage, and combination with anti-emetics to enhance lethality [12, 33–38]. A study of one such forum, the *Sanctioned Suicide* website, found a significant increase in sodium nitrite-related posts, strongly correlating with real-life suicides as evidenced by CDC and *National Poison Data System* reports, with the site accumulating over 10 million page views in September 2022 and surpassing 30,000 members by March 2023 [12]. This platform openly discusses methods of suicide, circumventing the restrictions imposed by larger social media sites, underscoring the need for targeted regulatory and public health interventions [12,36]. Despite recent restrictions by marketplaces such as *Etsy* and *eBay*, sodium nitrite remains widely accessible in high-purity forms (98–99%) from other vendors [12,36]. Lawsuits against *Amazon* in 2022 and 2023 alleged that the platform indirectly provided ‘suicide kits’ by enabling the sale of sodium nitrite [36]. Minimal regulation in many countries exacerbates the risks, permitting the purchase of potentially lethal quantities of sodium nitrite without oversight. A tragic case involved a 13-year-old girl who ordered sodium nitrite online and was later found deceased, with elevated nitrite levels detected in her gastric contents during postmortem analysis. Such incidents highlight the urgent need for stricter regulatory measures [37]. Regulations concerning sodium nitrite vary significantly across regions, offering valuable insights into effective strategies. In the UK, sodium nitrite has been designated as a ‘reportable substance’, requiring retailers to report suspicious purchases to law enforcement, thereby enhancing oversight and reducing misuse [36]. Canada has also taken targeted enforcement actions, as exemplified by the 2023 prosecution of an individual for distributing sodium nitrite online, demonstrating the deterrent effect of legal measures [36]. In the European Union (EU), Regulation (EU) 2023/2108 strengthens nitrite and nitrate regulations by reducing the maximum allowable levels of nitrites in food to mitigate health risks, including carcinogenic nitrosamine formation, while ensuring microbiological safety; it also sets maximum residual levels of nitrites and nitrates for food products at the time of market placement and throughout shelf life so that to enhance consumer exposure monitoring [39]. Denmark has adopted an even more precautionary approach, maintaining lower maximum nitrite levels than the broader EU standards and disallowing the marketing of products based solely on residual levels [39]. In contrast, the United States has less restrictive regulations, with the FDA permitting sodium nitrite for food preservation at concentrations up to 200 parts per million (ppm) in finished meat products and 500 ppm for sodium nitrate, while its widespread availability for non-food purposes through online marketplaces remains largely unregulated [40]. Thus, as substantiated by global epidemiological studies, sodium nitrite-related suicides remain a growing concern, with the U.S. *National Violent Death Reporting System* (NVDRS) documenting 260 cases from 2018 to 2020 as incidence rates increased from 0.01 to 0.09 per 100,000 person-years, primarily among young white males with depressive disorders [9]. Similar trends have been reported in Canada, Australia, and Europe, where its use as a means of suicide is also increasingly prevalent among younger individuals [14–18]. The promotion of sodium nitrite in euthanasia-support networks and publications such as *The Peaceful Pill Handbook* further compounds the issue. These resources present sodium nitrite as an effective, affordable, and accessible method for suicide, enhancing its appeal [11,33]. In summary, the increasing use of sodium nitrite for suicide underscores the need for coordinated public health measures, including international cooperation, stricter regulations, improved monitoring of online sales, enhanced reporting requirements, and public awareness campaigns, while healthcare and forensic practitioners must remain vigilant in identifying and addressing sodium nitrite poisoning with the objective to mitigate its impact.

Methemoglobinemia, the hallmark condition of nitrite toxicity, results from elevated levels of methemoglobin (MetHb). This condition impairs oxygen transport by converting hemoglobin’s ferrous iron (Fe^2+^) to ferric iron (Fe^3+^), rendering it unable to bind oxygen. Under physiological conditions, MetHb levels remain below 1%–2%, but concentrations exceeding this threshold cause systemic symptoms such as cyanosis, tachycardia, and lethargy. Severe cases, where MetHb levels surpass 50%–70%, can lead to arrhythmias, seizures, and coma, with levels above 70% often proving fatal. However, survival has been documented in exceptional cases with aggressive medical intervention [41–44]. Clinical and forensic recognition of methemoglobinemia relies on hallmark findings such as chocolate-brown blood, central cyanosis, and hypoxia that does not improve with oxygen therapy, emphasizing the importance of rapid and accurate diagnosis [41–43]. Epidemiological studies highlight the increasing prevalence of nitrate and nitrite-induced methemoglobinemia. For example, a retrospective review of the *National Poison Data System* (NPDS) records in the United States identified over 1,200 cases requiring methylene blue treatment, with nitrates and nitrites accounting for a significant proportion [45]. This rise underscores the need for enhanced regulation, accurate labeling, and improved public awareness so that to mitigate associated health risks. The lethal dose of sodium nitrite in adults varies widely, ranging from 0.7 to 6 g, with survival documented following ingestion of 6,000 mg under aggressive medical intervention. However, doses as low as 2.6 g – which is equivalent to the therapeutic dose for cyanide poisoning – are potentially fatal. Symptoms can appear acutely within 20 minutes to 3 hours after ingesting 200–500 mg, as demonstrated in both human and animal studies [8,33,46]. Treatment for methemoglobinemia involves prompt intravenous administration of methylene blue at a dose of 1–2 mg/kg over 5 minutes, rapidly reducing methemoglobin to hemoglobin and leading to clinical improvements within minutes [47–49]. For severe cases unresponsive to methylene blue, hyperbaric oxygen therapy can serve as an adjunctive treatment, providing rapid tissue oxygenation independent of MetHb levels [50]. In forensic toxicology, precise detection and quantification of nitrite and nitrate are critical for diagnosing sodium nitrite/nitrate toxicity. Nitrite stability in whole blood is preserved for up to 30 days at -20°C when stabilized with potassium ferricyanide (6.6 g/L), enabling long-term forensic analysis by preventing hemoglobin oxidation [51]. Accurate diagnosis of nitrite/nitrate toxicity requires MetHb measurement, quantification of nitrite and nitrate across multiple matrices, and identification of hypoxia markers to confirm respiratory failure as a cause of death, while storage artifacts affecting MetHb levels necessitate immediate analysis upon sample arrival to prevent misinterpretation of pre-mortem values [6,52]. However, in our study, MetHb levels were not obtained during the investigation, which represents a limitation. While other findings, such as systemic hypoxia, cherry-brown discoloration, and toxicological evidence, strongly suggest methemoglobinemia, quantitative MetHb data would have provided more direct evidence of oxygen transport impairment. Clinically, sodium nitrate toxicity can cause pseudohyperchloremia due to nitrate interference in analyzers, with a study demonstrating this by validating the Alinity analyzer (Abbott), which utilizes a silver chloride redox electrode system, as a more accurate method [53]. Elevated nitrate levels in serum and gastric contents serve as key forensic markers of sodium nitrite/nitrate intoxication, typically exceeding endogenous levels, while alternative matrices like costal cartilage and vitreous humor can further enhance the postmortem detection of nitrites and nitrates [54–56]. Rapid presumptive identification of nitrites can be achieved by using Griess reagent test strips, such as *MQuant™ Nitrite Test Strips*, which provide a cost-effective diagnostic tool in vitreous humor. However, the Griess method’s variability limits its precision compared to more reliable techniques like mass spectrometry [5,6,46,54]. In contrast, *Ion Chromatography* (IC) provides a highly sensitive (0.4 μmol/L detection limit) and specific method for distinguishing toxic nitrite/nitrate levels from baseline levels. IC modifications also overcome chloride interference, enabling accurate nitrite/nitrate measurement in postmortem toxicology, where nitrite oxidizes rapidly to nitrate [57,58]. Due to the lack of accessible and validated methods for direct nitrate analysis in forensic laboratories, MetHb measurement by spectrophotometry remains the standard for diagnosing nitrite/nitrate poisoning [6]. While conventional CO-oximeters for detecting MetHb and carboxyhemoglobin (COHb) are expensive and impractical in resource-limited settings, emerging technologies like the SCiO pocket molecular scanner offer a promising, affordable alternative by utilizing infrared spectroscopy for accurate on-site dyshemoglobin detection [59]. Advanced analytical techniques, such as capillary ion analysis (CIA) with UV detection and mass spectrometry, also provide reliable precision in nitrite/nitrate quantification, overcoming the limitations of older methods like the Griess assay [5,6,46].

Autopsy findings in nitrate-related fatalities, particularly those involving sodium nitrite, consistently reveal hallmark asphyxial signs, including scleral congestion, cyanosis of extremities, and distinctive multi-colored livor mortis. These range from gray to blue, brown, or purple and are most prominently observed on the face, hands, and shoulders [7,17,34,35]. The presence of chocolate-brown blood, which is a key indicator of sodium nitrite poisoning and methemoglobinemia, appears variably across cases, potentially due to differing methemoglobin levels at the time of death [7,17,35]. Histopathological analyses commonly show generalized visceral congestion and pulmonary edema, features consistent with severe hypoxic injury. The lungs frequently exhibit extensive swelling, marked vascular stasis, and, in some cases, haemorrhagic effusions under the pleura [7,17,34,35]. These findings mirror those observed in the current case report, where pulmonary edema with marked vascular stasis was noted, emphasizing the hypoxic damage typically associated with sodium nitrite ingestion. Additionally, cerebral edema with flattened gyri and numerous petechial hemorrhages were identified, similar to previously reported cases of nitrite-induced hypoxia [17,35]. A striking blue-gray or gray-brown cyanosis of the nail beds and skin provides another hypoxic marker indicative of elevated methemoglobin levels. The reported case presented cherry-brown lividity, consistent with methemoglobinemia, supporting findings documented in the literature. Notably, grayish-purple hypostasis has also been observed in some cases, highlighting variability in external findings depending on the extent of nitrite exposure and post-mortem intervals [7,17]. Internal examination typically reveals brain swelling, subpleural petechiae, and increased blood flow across internal organs, accompanied by characteristic dark brown or red-brown coloration of the blood and organs. These systemic nitrite-related changes were evident in the presented case and align with prior findings in sodium nitrite-related fatalities [17,35]. The death of the 19-year-old female described in this report highlights findings suggestive of intentional sodium nitrate (possibly saltpeter) and sodium nitrite ingestion, alongside multiple medications and alcohol. Post-mortem examination revealed cherry-brown lividity and markers of hypoxia, including pulmonary edema, cerebral edema, and dark, non-coagulated blood within the heart chambers. These findings closely parallel those documented by other authors [7,17,34,35], underscoring the typical systemic effects of nitrite toxicity. Toxicological analysis identified a blood alcohol concentration of 1.22‰, in addition to trace levels of amphetamine, atropine, and quetiapine, with nitrites detected in the gastric contents but absent in the intestines. These findings indicate multi-organ failure due to the toxic synergistic effects of sodium nitrate, nitrites, alcohol, and pharmaceuticals. Similar cases have demonstrated the combined impact of nitrites and co-ingested substances, where toxicological profiles reveal significant contributions to the fatal outcome [17,34,35]. Given the nonspecific nature of some autopsy findings, combining post-mortem examination results with toxicological analysis, scene investigation, and medical history assessment is essential to confirm sodium nitrite intoxication as the immediate cause of death [7,17,34,35]. This approach ensures comprehensive forensic evaluation and facilitates accurate determination of the factors contributing to mortality.

## Conclusions

In conclusion, nitrate and nitrite toxicity poses significant health risks, particularly in cases of intentional ingestion of sodium nitrite. While nitrates are generally less toxic, their conversion to nitrites can cause methemoglobinemia, a severe condition characterized by impaired oxygen transport, cyanosis, and hypoxia. Sodium nitrite, widely used in food preservation and industry, has become increasingly associated with suicide cases due to its easy availability and the dissemination of lethal dosing guidance online. This highlights the urgent need for stricter regulations and enhanced public awareness to address the misuse of this substance. This case report presents the fatal outcome of multi-substance ingestion in a 19-year-old female in Lithuania, as investigated by the Lithuanian State Forensic Medicine Service. Key findings include nitrite-induced hypoxia, systemic organ congestion, and characteristic cherry-brown discoloration of post-mortem lividity, which are hallmark signs of methemoglobinemia. While forensic toxicology analysis confirmed nitrite exposure, the absence of direct methemoglobin (MetHb) quantification represents a limitation, as such measurements would provide valuable insights into the severity of the poisoning and its contribution to the fatal outcome. The increasing availability of sodium nitrite for misuse underscores the necessity of preventive strategies. These include regulating its distribution, restricting access through online marketplaces, and monitoring content that promotes its misuse. Furthermore, public education campaigns are critical in raising awareness of the risks associated with sodium nitrite toxicity and reducing the incidence of intentional poisonings.
